# Supplementary material for: Identification of photoperiod-induced specific miRNAs in the adrenal glands of Sunite sheep (Ovis aries)
Source: Front Vet Sci. 2022 Jul 22;9:888207. doi: 10.3389/fvets.2022.888207 (PMC9354845; doi:10.3389/fvets.2022.888207)
Supplement: Supplementary file 1 [file Data_Sheet_1.ZIP › Supplementary file/miRDeep2 file.zip!/miRDeep2 file/Novel_Structure/SPLP42A3/Novel_664.pdf]

Provisional ID	:	NC_019481.2_36264
Score total	:	374.7
Score for star read(s)	:	3.9
Score for read counts	:	367.2
Score for mfe	:	2
Score for randfold	:	1.6
Score for cons. seed	:	
Total read count	:	732
Mature read count	:	680
Loop read count	:	0
Star read count	:	52

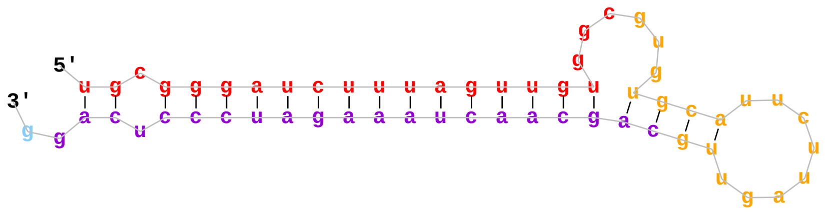

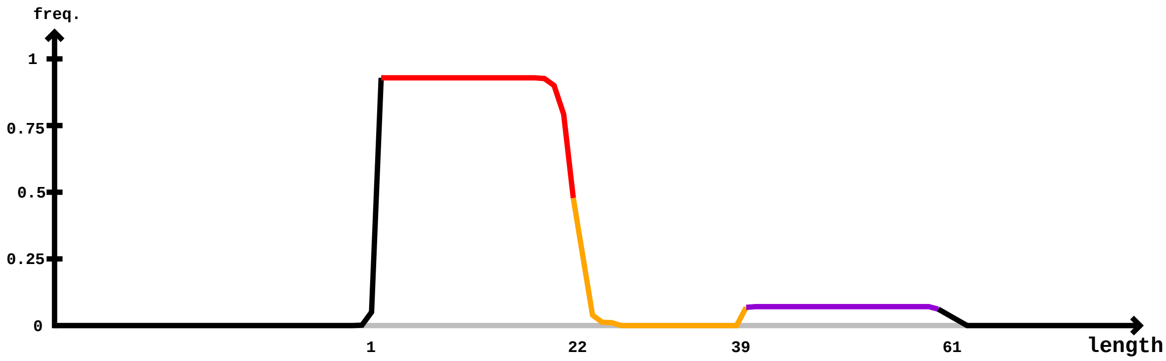

Mature   Star

```
5'- aauaacccauaaaaagcagccgacguugcggcaugcgggaucuuuaguuguggcgugugcauucuuaguugcagcaacuaaagaucccucaggcugcaaugaagaucagaga  -3'  obs
    aauaacccauaaaaagcagccgacguugcggcaugcgggaucuuuaguuguggcgugugcauucuuaguugcagcaacuaaagaucccucaggcugcaaugaagaucagaga        exp
    ....................(..(((((((((.((.(((((((((((((((......((((........))))))))))))))))))).)).)))))))))..)........  reads  mm  sample
    ...............................caugcgggaucuuuaguugugg...........................................................  1  0  seq
    ................................augcgggaucuuuaguugu.............................................................  5  0  seq
    ................................augcgggaucuuuaguugug............................................................  1  0  seq
    ................................augcgggaucuuuaguugugU...........................................................  1  1  seq
    ................................augcgggaucuuuaguugAgg...........................................................  1  1  seq
    ................................augcgggGucuuuaguugugg...........................................................  1  1  seq
    ................................augcgggaucuuuaguugugg...........................................................  9  0  seq
    ................................augcgggaucuuuaguuguggU..........................................................  13  1  seq
    ................................augcUggaucuuuaguuguggc..........................................................  1  1  seq
    ................................augAgggaucuuuaguuguggc..........................................................  3  1  seq
    ................................augcCggaucuuuaguuguggc..........................................................  1  1  seq
    .................................ugcgggaucuuuaguuA..............................................................  2  1  seq
    .................................ugcCggaucuuuaguugu.............................................................  1  1  seq
    .................................ugcgggaucuuuaguugC.............................................................  13  1  seq
    .................................ugcgggaucuuuaguuguA............................................................  22  1  seq
    .................................uAcgggaucuuuaguugug............................................................  1  1  seq
    .................................ugcgggGucuuuaguugug............................................................  1  1  seq
    .................................ugcAggaucuuuaguugug............................................................  1  1  seq
    .................................ugcgggaucuuuaguugug............................................................  52  0  seq
    .................................ugcgAgaucuuuaguugug............................................................  1  1  seq
    .................................uAcgggaucuuuaguugugg...........................................................  1  1  seq
    .................................ugcCggaucuuuaguugugg...........................................................  1  1  seq
    .................................ugcgggaucuuuaguugugA...........................................................  188  1  seq
    .................................ugcUggaucuuuaguugugg...........................................................  1  1  seq
    .................................ugcgggaucuuuaguuguAg...........................................................  1  1  seq
    .................................ugcgggaucuuuaguugugU...........................................................  25  1  seq
    .................................ugcCggaucuuuaguuguggc..........................................................  8  1  seq
    .................................ugcgggaucuuuaguuguggU..........................................................  249  1  seq
    .................................ugcgggaucuuuaguuguAgc..........................................................  1  1  seq
    .................................ugcgggaucuuuaguugAggc..........................................................  1  1  seq
    .................................ugcgggaucuuuaguugCggc..........................................................  1  1  seq
    .................................uAcgggaucuuuaguuguggc..........................................................  6  1  seq
    .................................ugUgggaucuuuaguuguggc..........................................................  2  1  seq
```

```
                                 Mature                               Star
aauaacccauaaaaagcagccgacguugcggcaugcgggaucuuuaguuguggcgugugcauucuuaguugcagcaacuaaagaucccucaggcugcaaugaagaucagaga
.................................ugcgggGucuuuaguuguggcg.........................................................   6    1    seq
.................................uAcgggaucuuuaguuguggcg.........................................................   10   1    seq
.................................ugAgggaucuuuaguuguggcg.........................................................   1    1    seq
.................................ugcUggaucuuuaguuguggcg.........................................................   3    1    seq
.................................ugcgggaucuuuagCuguggcg.........................................................   3    1    seq
.................................ugcgAgaucuuuaguuguggcg.........................................................   12   1    seq
.................................ugcUggaucuuuaguuguggcgu........................................................   4    1    seq
.................................ugcgggGucuuuaguuguggcgu........................................................   3    1    seq
.................................ugcgggaucuuuaguuguggUgu........................................................   4    1    seq
.................................uAcgggaucuuuaguuguggcgu........................................................   8    1    seq
.................................ugcgggaucuuuagCuguggcgu........................................................   1    1    seq
.................................ugcgggGucuuuaguuguggcgug.......................................................   1    1    seq
.................................ugcgggaucuuuaguuguggcgugu......................................................   8    0    seq
.......................................................................cCgcaacuaaagaucccuca.....................   5    1    seq
.......................................................................cagcaacuaaagaucccucag....................   45   0    seq
........................................................................agcaacuaaagaucccuca.....................   2    0    seq
```
